# Regulation of the JNK pathway by TGF-beta activated kinase 1 in rheumatoid arthritis synoviocytes

**DOI:** 10.1186/ar2215

**Published:** 2007-06-08

**Authors:** Deepa R Hammaker, David L Boyle, Tomoyuki Inoue, Gary S Firestein

**Affiliations:** 1 Division of Rheumatology, Allergy and Immunology, UCSD School of Medicine, Gilman Dr., La Jolla, CA 92093, USA; 2 Medicinal Research Laboratories, Taisho Pharmaceutical Co. Ltd, Yoshino-Cho, Kita-Ku, Saitama 331-9530, Japan

## Abstract

c-Jun N-terminal kinase (JNK) contributes to metalloproteinase (MMP) gene expression and joint destruction in inflammatory arthritis. It is phosphorylated by at least two upstream kinases, the mitogen-activated protein kinase kinases (MEK) MKK4 and MKK7, which are, in turn, phosphorylated by MEK kinases (MEKKs). However, the MEKKs that are most relevant to JNK activation in synoviocytes have not been determined. These studies were designed to assess the hierarchy of upstream MEKKs, MEKK1, MEKK2, MEKK3, and transforming growth factor-β activated kinase (TAK)1, in rheumatoid arthritis (RA). Using either small interfering RNA (siRNA) knockdown or knockout fibroblast-like synoviocytes (FLSs), MEKK1, MEKK2, or MEKK3 deficiency (either alone or in combination) had no effect on IL-1β-stimulated phospho-JNK (P-JNK) induction or MMP expression. However, TAK1 deficiency significantly decreased P-JNK, P-MKK4 and P-MKK7 induction compared with scrambled control. TAK1 knockdown did not affect p38 activation. Kinase assays showed that TAK1 siRNA significantly suppressed JNK kinase function. In addition, MKK4 and MKK7 kinase activity were significantly decreased in TAK1 deficient FLSs. Electrophoretic mobility shift assays demonstrated a significant decrease in IL-1β induced AP-1 activation due to TAK1 knockdown. Quantitative PCR showed that TAK1 deficiency significantly decreased IL-1β-induced MMP3 gene expression and IL-6 protein expression. These results show that TAK1 is a critical pathway for IL-1β-induced activation of JNK and JNK-regulated gene expression in FLSs. In contrast to other cell lineages, MEKK1, MEKK2, and MEKK3 did not contribute to JNK phosphorylation in FLSs. The data identify TAK1 as a pivotal upstream kinase and potential therapeutic target to modulate synoviocyte activation in RA.

## Introduction

Rheumatoid arthritis (RA) is a chronic inflammatory disease characterized by synovial lining hyperplasia and sublining infiltration of inflammatory cells [1]. Fibroblast-like synoviocytes (FLSs) play a crucial role in joint damage as well as the propagation of inflammation [2]. In response to potent pro-inflammatory cytokines such as IL-1β, FLSs produce large amounts of matrix metalloproteinases (MMP), which are key drivers of matrix destruction [3-5]. MMP production is, in turn, regulated by several signal transduction pathways, including the mitogen-activated protein kinases (MAPKs) [6,7].

All three MAPK families have been implicated in RA, including extracellular signal-regulated kinase (ERK), c-Jun N-terminal kinase (JNK), and p38 [8-10]. JNK plays an especially important role in extracellular matrix turnover because it is activated in RA synovium, regulates MMP gene expression in cultured FLSs, and mediates joint destruction in rat adjuvant arthritis [11-16]. JNK is phosphorylated by upstream MAPK kinases (MAPKKs), which are dual specific enzymes that phosphorylate threonine and tyrosine residues [17]. Two MAPKKs (or mitogen-activated protein kinases [MEKs]), MKK4 and MKK7, form a complex with JNK [18], although only the latter is required for cytokine-mediated engagement of this pathway in FLSs [19].

Multiple upstream MAPKK kinases (MAP3Ks) that activate the MAPKKs and the JNK cascade have been identified in RA. For instance, MEK kinase (MEKK)1, MEKK2, and transforming growth factor-β activated kinase (TAK)1 are the most abundant in inflamed synovium as well as cultured FLSs [20]. Of these MAP3Ks, MEKK2 initially appeared to be the most important in RA because it forms a functional complex with JNK. In the present study, TAK1 functioned as the dominant MAP3K for JNK activation in IL-1-stimulated FLSs. These results were unexpected because several groups have shown that MEKK1, MEKK2 and MEKK3 are indispensable for JNK activation. For instance, MEKK1 is the predominant kinase required for JNK activation in corneal epithelia [20] and murine embryonic fibroblasts (MEFs) [20]. In other culture conditions, JNK activation is inhibited in MEKK3-/- MEFs stimulated with IL-1 [21]. Similarly, fibroblast growth factor (FGF)-2-induced JNK activation and JNK phosphorylation-induced T cell receptor ligation require MEKK2 [22]. Based on our studies using MAP3K deficient cells, these MAP3Ks appear to be redundant in JNK activation in cultured FLSs. Therefore, the diverse and complex functions of MAP3Ks vary depending on the cell type as well as the stimulus. It is precisely this signaling diversity that offers an opportunity to target upstream kinases in the JNK cascade that regulate pathogenic responses in arthritis while potentially sparing other functions that are critical to host responses. This study suggests that TAK1 is a crucial activator of the JNK pathway in FLSs and is a potential target for arthritis therapy.

## Materials and methods

### Fibroblast-like synoviocytes

FLSs were isolated from synovial tissues obtained from RA patients at the time of joint replacement as described previously [3]. The diagnosis of RA conformed to the American College of Rheumatology 1987 revised criteria [23]. The protocol was approved by the UCSD Human Subjects Research Protection Program. Synovial tissues were minced and incubated with 0.5 mg/ml collagenase VIII (Sigma, St. Louis, MO, USA) in serum-free RPMI (Mediatech, Herndon, VA, USA) for 1.5 h at 37°C, filtered through a 0.22 μm cell strainer, extensively washed, and cultured in DMEM supplemented with 10% FCS (endotoxin content <0.006 ng/ml; Gemini Biosciences, Calabasas, CA, USA), penicillin, streptomycin, gentamicin and L-glutamine in a humidified 5% CO_2 _incubator. After overnight culture, nonadherent cells were removed, and adherent cells were trypsinized, split at a 1:3 ratio, and cultured. Synoviocytes were used from passage 4 through 9, when FLSs were a homogeneous population with <1% CD11b, <1% phagocytic, and <1% FcRγII positive cells.

Mice knee and ankle synovial tissues were isolated, minced and incubated with 0.5 mg/ml collagenase VIII (Sigma) in serum-free RPMI (Mediatech) for 1.5 h at 37°C, extensively washed, and cultured in DMEM supplemented with 10% FCS (endotoxin content <0.006 ng/ml; Gemini Biosciences), penicillin, streptomycin, gentamicin and L-glutamine in a humidified 5% CO_2 _incubator. After three days of culture, non-adherent cells were removed, and adherent cells were trypsinized, split at a 1:3 ratio, and cultured. Synoviocytes were then used from passage 4 through 9.

### Antibodies and reagents

Affinity purified rabbit polyclonal MEKK1, MEKK2, mouse monoclonal TAK1, mouse monoclonal GAPDH, goat polyclonal actin antibodies and secondary antibodies were purchased from Santa Cruz Biotechnology (Santa Cruz, CA, USA). Rabbit polyclonal phospho-JNK (P-JNK), P-p38, P-ERK, P-MKK4, P-MKK7, JNK, and secondary horse-raddish peroxidase (HRP)-conjugated antibodies and GST-c-Jun were purchased from Cell Signaling Technology (Danvers, MA, USA). Anti-MEKK3, MKK4, MKK7, and appropriate secondary antibodies were purchased from Upstate Biotechnology (Lake Placid, NY, USA). rhIL-1β was purchased from R&D Systems (Minneapolis, MN, USA).

### Fibroblast-like synoviocyte transfection

Using the Amaxa Human Dermal Fibroblast Nucleofector kit (NHDF-adult) with program U-23, 2 to 5 × 10^5 ^cells (passages 4 to 6) were transfected with 1 to 5 μg of MEKK1, MEKK2, MEKK3, TAK1, or scrambled (sc) negative control Smartpool small interfering RNA (siRNA; Dharmacon, Lafayette, CO, USA), according to the manufacturer's protocol (Amaxa, Gaithersburg, MD, USA) [19].

### Western blot analysis

After transfection, FLSs were cultured in DMEM with 10% FCS in six-well plates for appropriate times and synchronized in DMEM with 0.1% FCS. FLSs were then treated with medium or rhIL-1β (2 ng/ml; R&D Systems) for 15 minutes. Cell lysates were obtained as described previously [19]. Whole cell lysates (50 μg) were fractionated on Tris-glycine-buffered 10% SDS-PAGE and transferred to nitrocellulose membrane (Biorad, Hercules, CA, USA). The membranes were blocked with 5% nonfat milk in 0.05% Tween 20/Tris-buffered saline(TBS) for 1 h at room temperature, followed by incubation with primary antibody (1:1000) overnight at 4°C. The blots were then incubated in the secondary antibody for 2 h at room temperature. Immunoreactive protein was detected with enhanced chemiluminescence (Perkin Elmer, Waltham, MA, USA) and autoradiography, which was analyzed using NIH Image (version 1.63) and normalized to actin or GAPDH expression.

### Immunoprecipitation and kinase assays

siRNA-transfected FLSs were stimulated with either medium or IL-1 (2 ng/ml) and lysed at appropriate times, as previously described [19]. The lysate (100 μg) was then incubated with anti-JNK, anti-MKK4, or anti-MKK7 antibodies (2 μg) for 4 h at 4°C on a rotator, followed by incubation with protein A-agarose overnight. The immunoprecipitates were washed and resuspended in 25 μl of kinase buffer (50 mM HEPES, pH 7.4, 1 mM MgCl_2_, 20 mM β-glycerophosphate, 1 mM Na_3_VO_4_, 0.2 mM dithiothreitol, 10 μg/ml aprotinin, 1 μM pepstatin A, and 1 mM phenylmethylsulfonyl fluoride) containing 5 mCi of [γ-^32^P]-ATP, 25 μM ATP, and 8 μg of GST-c-Jun, and incubated at 37°C for 30 minutes. Reactions were stopped with 5 μl of 6× SDS sample buffer (100 mM Tris, pH 6.8, 2% SDS, 10% glycerol, 5% 2-ME, 0.25% bromophenol blue). After electrophoresis and autoradiography, the data were analyzed using NIH Image (version 1.63).

### Electrophoretic mobility shift assay

Following transfection, FLSs were seeded in 10 cm dishes and cultured in DMEM with 10% FCS at 37°C for 24 h. The cells were incubated in fresh media for 48 h and subsequently serum starved (0.1% FCS/DMEM) for 48 h. FLSs were then treated with either medium or IL-1β (2 ng/ml) for 60 minutes. The cells were rinsed twice with phosphate-buffered saline and nuclear extracts were isolated using a nuclear protein extraction kit (Chemicon, Temecula, CA, USA) and protein estimation was performed using the micro-BCA kit (Pierce, Rockford, IL, USA). Nuclear extracts (10 μg) were incubated with [γ-^32^P]-ATP labeled or unlabeled AP-1 (5'-CGCTTGATGAGTCAGCCGGAA-3'), nuclear factor (NF)-κB (5'-AGTTGAGGGGACTTTCCCAGGC-3') oligonucleotides (Promega, Madison, WI, USA) for 20 minutes at room temperature and run on a 5% acrylamide/Tris-base EDTA (TBE) gel for 25 minutes at 200 V. The gel was dried and exposed to film. The autoradiograph was analyzed using NIH Image (version 1.63).

### IL-6 ELISA

After transfection, FLSs were seeded in 12-well plates and cultured in DMEM with 10% FCS at 37°C for 24 h. The supernatants were aspirated and replaced with fresh medium for 24 h. FLSs were then treated with medium or rhIL-1β (2 ng/ml) for 24 h and the supernatants were harvested. Samples were assayed for IL-6 by ELISA (R&D Systems).

### Quantification of MMP mRNA in FLS

mRNA from cultured FLSs was isolated using RNA-STAT (Tel-Stat, Friendswood, TX, USA) and cDNA was prepared, according to the manufacturer's instructions using GeneAmp 2400 (Applied Biosystems, Foster City, CA, USA). Quantitative real-time PCR was performed using Assays On Demand (Applied Biosystems) to determine relative mRNA levels using the GeneAmp 5700 Sequence Detection System (Applied Biosystems) as described previously [24]. Sample threshold cycle (Ct) values were used to calculate the number of cell equivalents in the test samples. The data were then normalized to GAPDH expression to obtain relative cell equivalents.

### Statistical analysis

Data are expressed as mean ± standard error of the mean. Comparisons between two groups were performed using Student's *t*-test. A comparison was considered statistically significant if *p *< 0.05.

## Results

### MAP3K knockdown by siRNA transfection in RA FLSs

**Figure 1:**
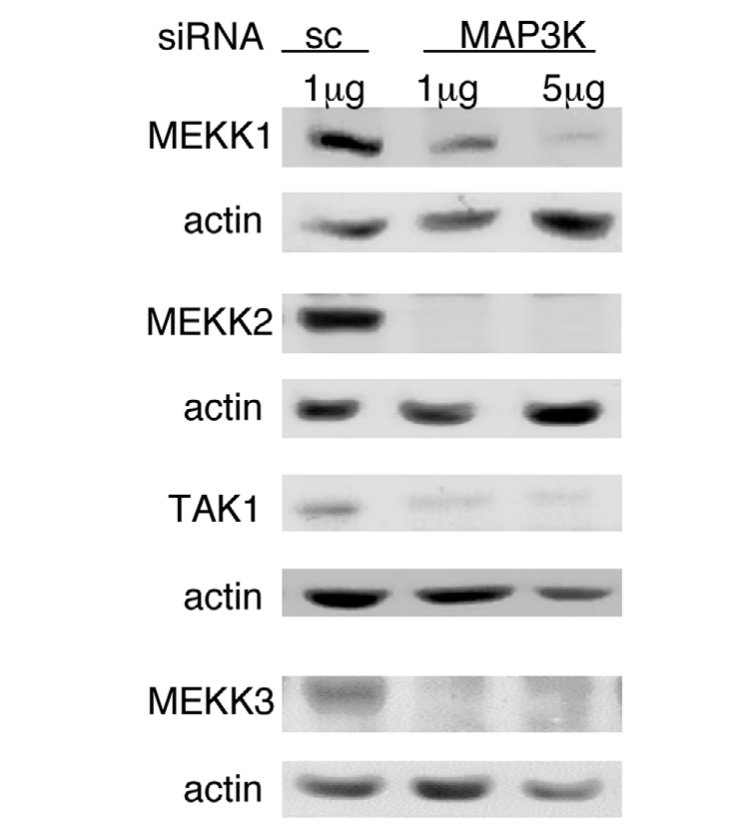
MAP3K knockdown by small interfering RNA (siRNA) in rheumatoid arthritis fibroblast-like synoviocytes (FLSs). Cultured FLSs were transfected with 1 or 5 μg of MEKK1, MEKK2, MEKK3, TAK1 or scrambled negative control (sc) siRNA as described in Materials and methods. FLSs were then incubated for 3, 5 and 7 days and western blot analysis was performed. siRNA specifically inhibited respective kinase expression by >75%. Optimal knockdown of MEKK1 (5 μg siRNA), MEKK2 (1 μg siRNA) and MEKK3 (1 μg siRNA) was observed on day 3 and on day 5 for TAK1 (1 μg siRNA).

To determine the optimal conditions for inhibiting MAP3K expression, FLSs were transfected with 1 or 5 μg of MEKK1, MEKK2, MEKK3, TAK1 or sc siRNA and lysates were prepared 3 to 5 days later. Western blot analysis was then performed using anti-MEKK1, -MEKK2, -MEKK3 and -TAK1 antibodies. As shown in Figure 1, each siRNA inhibited the respective kinase expression. Optimal inhibition of MEKK1 (5 μg siRNA), MEKK2 (1 μg siRNA) and MEKK3 (1 μg siRNA) expression was observed on day 3. TAK1 expression was inhibited using 1 μg siRNA on day 5.

### MEKK1, MEKK2 and MEKK3 knockdown do not alter IL-1-induced MAPK activation

**Figure 2:**
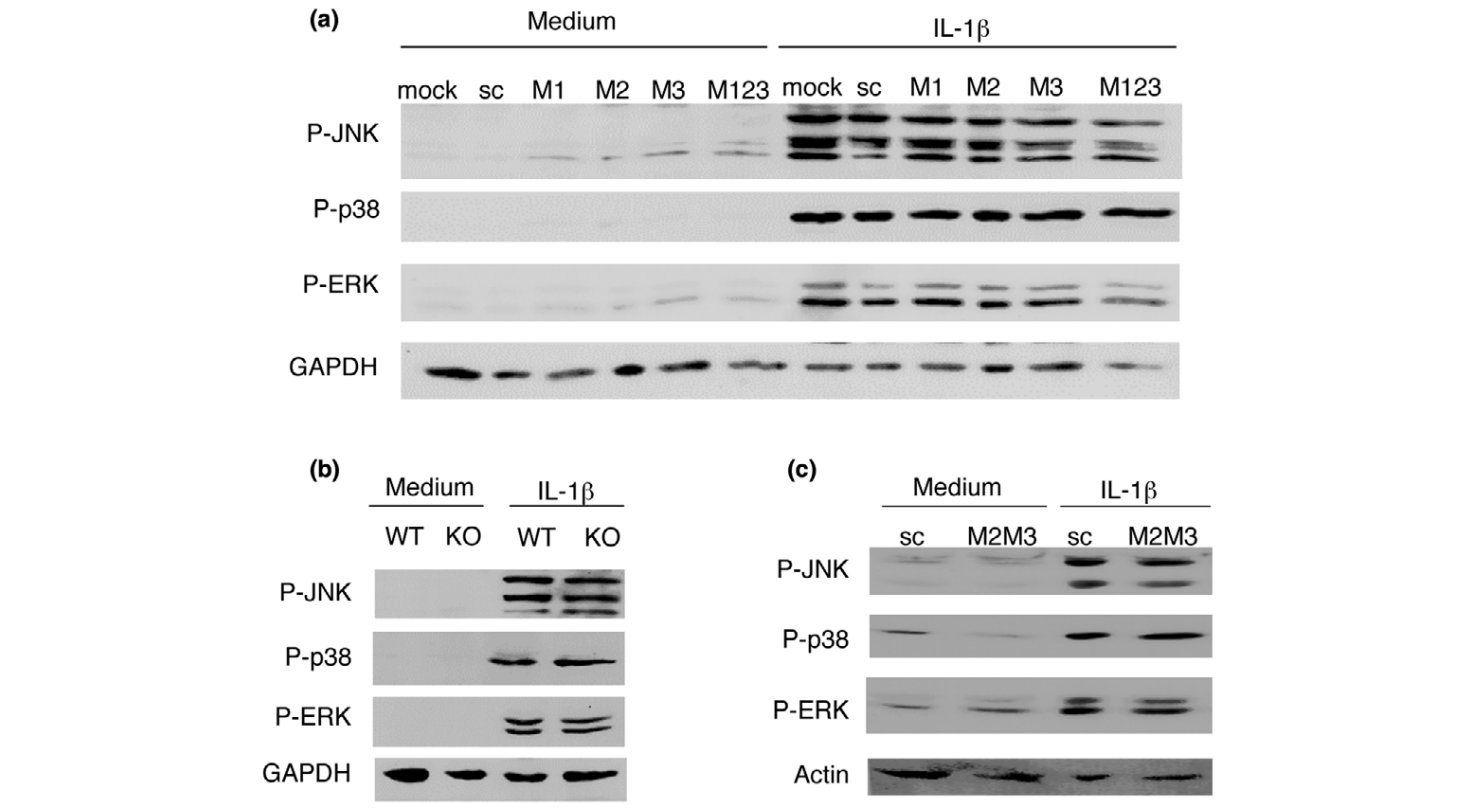
MEKK1, MEKK2 and MEKK3 do not alter IL-1β-induced mitogen-activated protein kinase (MAPK) activation. **(a) **Three days after small interfering RNA (siRNA) transfection, serum-starved fibroblast-like synoviocytes (FLSs) were stimulated with IL-1β (2 ng/ml) for 15 minutes and lysates were evaluated by western blot analysis. MEKK1, MEKK2, or MEKK3 deficiency alone or in combination had no effect on IL-1β-stimulated JNK, p38 or ERK activation compared with scrambled control (sc) (*n *= 2 separate FLS lines). M1, MEKK1 siRNA; M2, MEKK2 siRNA; M3, MEKK3 siRNA; M123, MEKK1+MEKK2+MEKK3 siRNA. **(b) **To complement the MEKK1 siRNA studies, MEKK1-/- FLSs were also examined. MEKK1 knockout (KO) and wild-type (WT) mouse FLSs were serum-starved for 48 h, stimulated with IL-1β (2 ng/ml) for 15 minutes and lysed. Cell extracts were evaluated by western blot analysis. MEKK1 deficiency did not affect IL-1β-induced MAPK activation. **(c) **MEKK1-/- mFLSs were transfected with MEKK2 and MEKK3 (M2M3) or sc siRNA, and, later, serum-starved for 48 h and stimulated with IL-1β (2 ng/ml) for 15 minutes. Western blot analysis of the lysates confirmed that MEKK2 and MEKK3 do not alter MAPK activation.

To determine the relative contributions of MEKK1, MEKK2, and MEKK3 to IL-1β-induced MAPK activation, FLSs were transfected with the corresponding siRNA, individually or in combination. On day 3, the serum starved FLSs were stimulated with IL-1β (2 ng/ml) and the lysates were evaluated by western blot analysis using anti-P-JNK, anti-P-p38, and anti-P-ERK antibodies. Figure 2a shows that MEKK1, MEKK2, and MEKK3 deficiency alone or in combination had no effect on IL-1-stimulated JNK, p38 or ERK activation. We then repeated the experiment using MEKK1-/- mouse FLS. Western blot analysis of IL-1β or medium treated MEKK1-/- FLS lysates confirmed data obtained from human FLSs transfected with MEKK1 siRNA (Figure 2b). Next, we transfected MEKK1-/- mouse FLS with MEKK2 and MEKK3 siRNA. IL-1β-induced JNK, p38, and ERK activation was then determined (Figure 2c). The results indicate that MEKK1, MEKK2 and MEKK3 deficiency do not alter the activation of JNK, p38 or ERK in IL-1β-stimulated FLSs.

### Effect of TAK1 knockdown on IL-1-induced JNK activation

**Figure 3:**
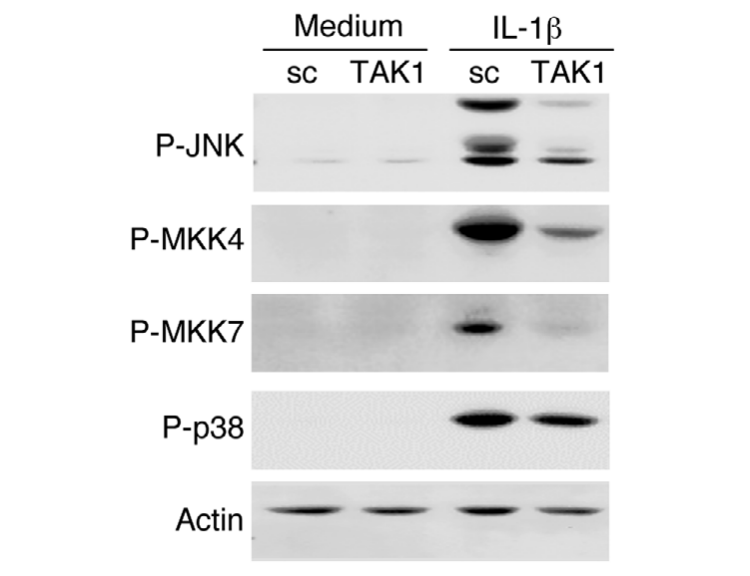
IL-1β-induced JNK activation in fibroblast-like synoviocytes (FLSs) is TAK1-dependent. The effect of TAK1 deficiency on JNK activation was determined by western blot analysis. Three days after TAK1 or scrambled control (sc) small interfering RNA transfection, serum-starved FLSs were stimulated with IL-1β (2 ng/ml) for 15 minutes. Cell lysates were evaluated for P-JNK, P-MKK4, P-MKK7, P-p38, and actin. JNK, MKK4, and MKK7, but not p38 activation was significantly decreased in the absence of TAK1 (average inhibition: JNK, 58 ± 1%, *p *= 0.01; MKK4, 49 ± 3%, *p *= 0.01; MKK7, 49 ± 7%, *p *= 0.04). A representative experiment is shown (*n *= 3).

**Figure 4:**
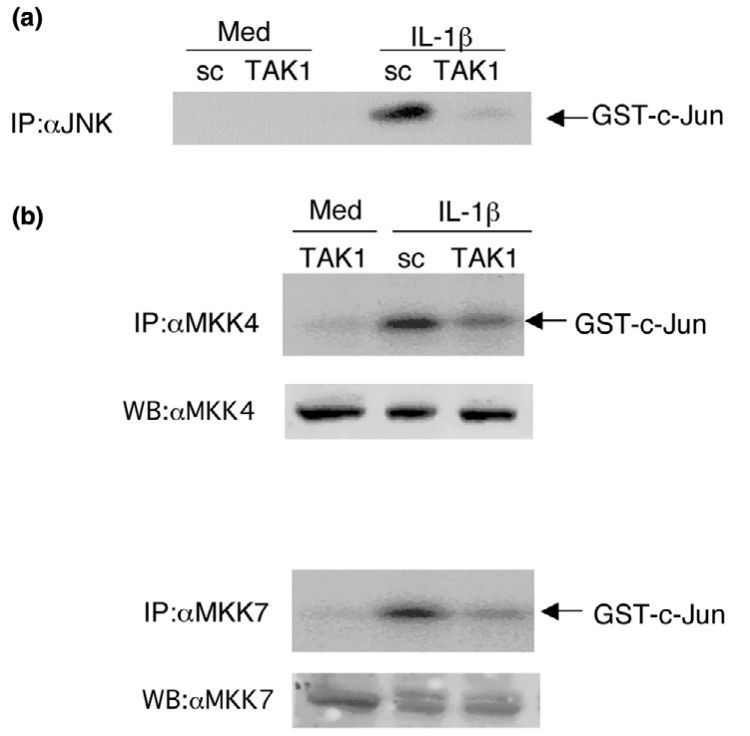
JNK kinase activity is TAK1-dependent. **(a) **Kinase assays were used to evaluate JNK function in TAK1 deficient cells. Fibroblast-like synoviocytes (FLSs) transfected with TAK1 or scrambled control (sc) small interfering RNA (siRNA) were serum-starved, stimulated with IL-1β (2 ng/ml) for 15 minutes, lysed, immunoprecipitated with anti-JNK antibodies, and subjected to kinase assay using GST-c-Jun substrate. JNK-mediated activation of c-Jun was significantly decreased in TAK1 deficient FLS (53 ± 2% inhibition, *p *= 0.03). A representative experiment is shown (*n *= 3). Med, medium; IP, immunoprecipitation. **(b) **Kinase assays were performed with anti-MKK4 and anti-MKK7 antibody immunoprecipitates and GST-c-Jun substrate to evaluate the effects of TAK1 deficiency on MKK4 and MKK7 function. Significant decreases in IL-1β-induced MKK4 and MKK7 kinase activity were observed (average inhibition: MKK4, 28 ± 4%, *p *= 0.004; MKK7, 28 ± 8%, *p *= 0.02). A representative experiment is shown (*n *= 3). Wb, Western blot.

To determine the effect of TAK1 knockdown on JNK and p38 activation, FLSs were transfected with TAK1 siRNA and then stimulated on day 5 with 2 ng/ml of rhIL-1β. As shown in Figure 3, TAK1 deficiency in FLSs significantly inhibited IL-1β-induced JNK, MKK4, and MKK7 phosphorylation compared with sc control (mean inhibition: JNK, 58 ± 1% (*p *= 0.01); MKK4, 49 ± 3% (*p *= 0.01); MKK7, 49 ± 7% (*p *= 0.04); *n *= 3 each). However, p38 activation was not affected by TAK1 deficiency. To determine the effect of TAK1 deficiency on JNK function, kinase assays were performed using anti-JNK, anti-MKK4, or anti-MKK7 antibodies and GST-cJun substrate (Figure 4a). GST-cJun phosphorylation by anti-JNK immunoprecipitates was significantly decreased in TAK1 deficient FLSs (53 ± 2% inhibition, *p *= 0.03, *n *= 3). In addition, TAK1 knockdown modestly inhibited MKK4 and MKK7 kinase activity (average inhibition: MKK4, 28 ± 4% (*p *= 0.01); MKK7, 28 ± 8% (*p *= 0.03); *n *= 3 each; Figure 4b).

### Regulation of AP-1 and NF-κB binding and transcriptional activity by TAK1

**Figure 5:**
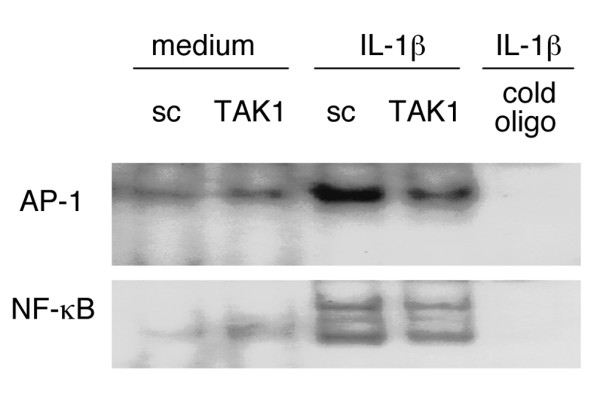
Regulation of AP-1 binding and transcriptional activity is TAK1-dependent. The effect of TAK1 deficiency on IL-1β-induced AP-1 activation was evaluated by electrophoretic mobility shift assay. Five days after small interfering RNA transfection, cultured fibroblast-like synoviocytes were stimulated with IL-1β (2 ng/ml) for 60 minutes. Nuclear extracts were obtained and evaluated for AP-1 and NF-κB binding activity. A representative experiment is shown (*n *= 3). IL-1β-induced AP-1 activity was significantly decreased in the absence of TAK1 (84 ± 8% inhibition compared to control, *p *= 0.03). NF-κB binding, on the other hand, was not significantly decreased with TAK1 deficiency (34 ± 7% inhibition, *p *= 0.21). Cold oligo, non-radioactive oligo; Sc, scrambled control.

AP-1 and NF-κB regulate MMP and pro-inflammatory cytokine gene expression by FLSs. To determine if TAK1 knockdown modulates AP-1 and NF-κB binding, electrophoretic mobility shift assays were performed (Figure 5). Similar levels of basal AP-1 and NF-κB binding were observed in control and TAK1 knockdown FLSs. AP-1 binding increased after IL-1β stimulation in the sc siRNA transfected lysates. However, TAK1 deficiency significantly inhibited IL-1β-induced AP-1 activation (84.3 ± 8.1% inhibition compared with control, *n *= 3, *p *= 0.028). There was a trend towards a decrease in NF-κB activation, although this did not reach statistical significance (34.2 ± 7.0% inhibition, *n *= 3, *p *= 0.21).

### Effect of TAK1 knockdown on MMP gene expression and IL-6 production

**Figure 6:**
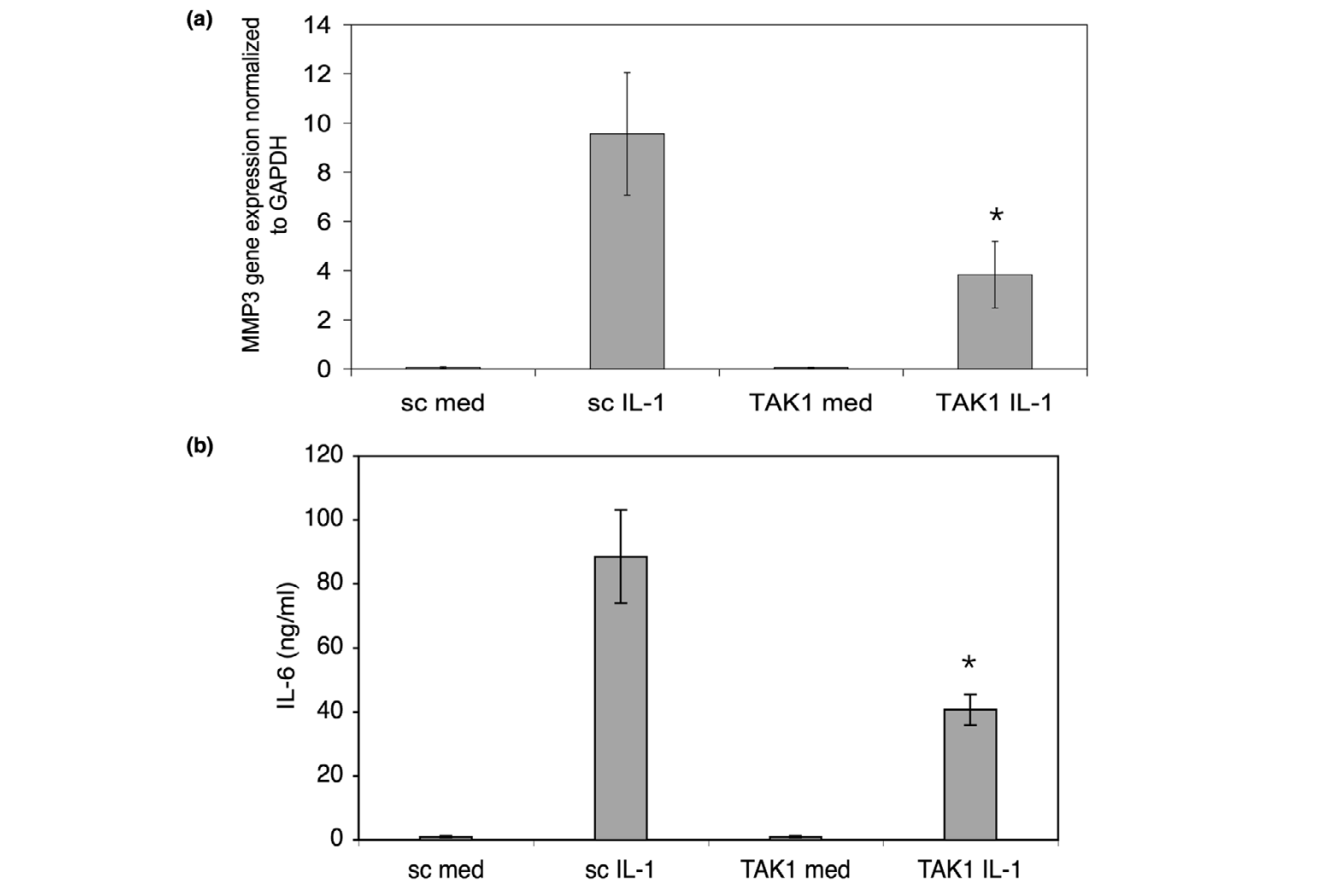
Effect of TAK1 knockdown on matrix metalloproteinase (MMP) gene expression and IL-6 production. **(a) **To determine if TAK1 deficiency affects MMP3 gene expression, real-time quantitative PCR was performed. TAK1 or scrambled control (sc) small interfering RNA-treated fibroblast-like synoviocytes (FLSs) were stimulated with IL-1β (2 ng/ml) for 24 h and MMP3 gene expression was assayed and normalized to GAPDH. Data are shown as relative expression units (REU). IL-1β-induced MMP3 gene expression in the absence of TAK1 was significantly decreased compared with sc control (56 ± 14% inhibition, *n *= 5, *p = 0.01). Med, medium. **(b) **The effect of TAK1 knockdown on IL-6 production was assayed by ELISA. Cell supernatants were collected from FLSs stimulated with IL-1β (2 ng/ml) for 24 h. TAK1-deficient FLSs produced significantly less IL-6 compared with sc control (53 ± 3% inhibition, *n *= 4, *p = 0.02).

Because TAK1 regulates IL-1β-induced AP-1 activation, we determined if TAK1 deficiency affects MMP3 gene expression by real-time quantitative PCR and IL-6 production by ELISA. TAK1 siRNA- or sc siRNA-treated FLSs were stimulated with IL-1β or medium for 24 h and assayed for MMP3 gene expression (Figure 6a). TAK1 deficiency significantly decreased IL-1β-induced MMP3 gene expression compared with sc control (GAPDH normalized average: 55.9 ± 14% inhibition, *n *= 5, *p *= 0.04). To measure IL-6 production, control or TAK1 knockdown FLSs were stimulated with IL-1β for 24 h. Cell supernatants were then collected and assayed by ELISA (Figure 6b). TAK1 deficient cells produced significantly less IL-6 compared with sc siRNA transfected cells (52.7 ± 3.3% inhibition, *n *= 4, *p *= 0.021).

## Discussion

RA is a chronic inflammatory disease of unknown etiology that targets the synovium. Intimal lining macrophages and fibroblast-like synoviocytes produce pro-inflammatory cytokines that contribute to synovial inflammation and production of destructive enzymes like MMPs [25]. The MMPs can then degrade components of the extracellular matrix, especially native interstitial collagen [26], initiating a series of events leading to irreversible joint damage [27]. MMP gene expression in FLSs is regulated by many signaling pathways, although MAPKs play a prominent role [9].

Of the three MAPK families, JNK is particularly interesting because it efficiently phosphorylates c-Jun. This protein is a crucial component of the transcription factor AP-1, which, in turn, initiates MMP gene expression [16,28]. JNK can be phosphorylated by two dual specificity threonine/tyrosine kinases, MKK4 and MKK7 [18]. Recent studies using siRNA to deplete individual kinases showed that MKK7, but not MKK4, is necessary for IL-1β-induced JNK activation in FLSs [19]. In contrast, both MKK4 and MKK7 are required for maximum JNK phosphorylation by anisomycin, lipopolysaccharide, and sorbitol [19,29].

MKK4 and MKK7 are, in turn, regulated by a large family of serine/threonine kinases known as the MAP3Ks that integrate extracellular stimuli and activate transcription factors in a cell-type and stimulus-specific manner [29]. Little is known about how MAP3Ks regulate the JNK pathway or MMP gene expression in RA. To address this issue, we previously examined MAP3K gene and protein expression in RA synovial tissue and FLSs [20]. These studies showed that four of the MAP3Ks, namely MEKK1, MEKK2, MEKK3 and TAK1, are abundant in RA FLSs. Kinase assays suggested that MEKK2 and TAK1 are especially important activators of the JNK pathway in FLSs.

In the present study, we examined the hierarchy of these proteins in IL-1β-mediated JNK activation in RA FLSs using siRNA. The results showed that MEKK1, MEKK2 and MEKK3 are not necessary for IL-1β-mediated JNK phosphorylation, either individually or in combination. The data also demonstrate that the pathways utilized by stress kinases can be cell and stimulus specific. For instance, MEKK1 is required for JNK and c-Jun activation in the corneal epithelia of MEKK1-/- mice [30]. MEKK1 is also critical for JNK activation in response to pro-inflammatory stimuli and cell migration in MEKK1-/- MEFs [31]. Unlike MEKK1, MEKK3 knockout is embryonic lethal [32] and JNK and p38 activation are defective in MEKK3-/- MEFs stimulated with IL-1β [21].

Several additional studies indicate that MEKK2 can play a cell-lineage specific role in JNK activation, and our previous studies showed that it could form a functional signaling complex in FLSs [20]. MEKK2 also associates with MKK7 and JNK1 in Cos cells [33]. Its role in JNK function was suggested by studies showing that MEKK2 gene disruption inhibits JNK activation in mast cells in response to c-Kit and Fcε RI stimulation [34]. Kesavan and colleagues showed that FGF-2-induced JNK activation also required MEKK2 in knockout MEFs [22]. Furthermore, MEKK2 is required for JNK activation in T cell receptor signaling and IL-2 gene expression [35]. Despite these data, siRNA and MEKK2-/- studies indicate that MEKK2 is not required for IL-1β-induced JNK activation in FLSs.

In contrast, TAK1 is a critical upstream kinase regulating JNK in FLSs. TAK1 is an evolutionarily conserved MAP3K that is essential for some innate and adaptive immune responses [36]. Signaling through the IL-1 receptor leads to ubiquination and activation of the tumor necrosis factor receptor-associated factor 6 (TRAF6)/TAB1/TAB2/TAB3 (TAB, TAK1-binding protein) complex through IL-1 receptor-associated kinase (IRAK) [37-41]. TAK1 is then activated by autophosphorylation of serine/threonine residues within its activation loop [42]. It can then engage I-kappaB kinase and MAPK pathways leading to the activation of NF-κB, p38, and JNK [43].

TAK1, unlike MEKK1, MEKK2, and MEKK3, is intimately involved in IL-1β-induced JNK activation in FLSs. TAK1 knockdown significantly inhibited the kinase activity of MKK4, MKK7, and JNK. However, TAK1 deficiency did not affect the p38 pathway or interferon gene expression (IP-10 and IFNβ). This result in FLSs differs from studies using 293 cells where p38 and JNK activation by IL-1β required TAK1 [44]. Once JNK is phosphorylated, its effect on downstream gene expression typically involves AP-1 activation. Transcription factor studies in FLSs confirmed that TAK1 deficiency not only decreased JNK activation but also suppressed AP-1 binding. The effect on NF-κB was less prominent and is consistent with previous studies in RANK ligand-stimulated 293 cells expressing dominant-negative TAK1 [45]. The variability of MAP3K function in different cell types is also underscored by studies implicating TAK1 in the NF-κB pathway in HeLa cells [46] and NIH3T3 cells [47]. Therefore, it is important to evaluate signaling mechanisms in tissue-specific cell lineages when considering their potential role in inflammatory diseases.

The functional consequences of activating the TAK1-JNK-AP1 pathway can be evaluated by determining expression of key AP-1-driven genes implicated in RA. The AP-1 consensus sequence is located at -70 base-pairs in the promoter region of the gene encoding MMP3, making it a useful biomarker for TAK1 in cells such as osteocytes [48] and chondrocytes [49]. siRNA studies showed that TAK1 inhibition significantly decreased MMP3 gene expression in cultured FLSs. Of interest, IL-1β-induced IL-6 production was also decreased by TAK1 deficiency, which could reflect an effect on AP-1 because this transcription factor also binds to the IL-6 promoter. The modest effect of TAK1 on NF-κB could also contribute to MMP3 and IL-6 expression.

## Conclusion

These data suggest that TAK1 is a key element in JNK activation, IL-6 production, and MMP expression by FLSs. Surprisingly, other MAP3Ks implicated in JNK activation, such as MEKK1, MEKK2, and MEKK3, do not have a major contribution to this pathway in FLSs. Therefore, targeting TAK1 might represent an alternative way to regulate JNK activation and matrix degradation in inflammatory arthritis.

## Abbreviations

Ct = threshold cycle; DMEM = Dulbecco's modeified Eagle's medium; ELISA = enzyme-linked immunosorbent assay; ERK = extracellular signal-regulated kinase; FCS = fetal calf serum; FGF = fibroblast growth factor; FLS = fibroblast-like synoviocyte; GAPDH = glyceraldehyde-3-phosphate dehydrogenase; GST = glutathione S-transferase; IL = interleukin; IRAK = IL-1 receptor-associated kinase; JNK = c-Jun N-terminal kinase; MAP3K = MAPKK kinase; MAPK = mitogen-activated protein kinase; MAPKK = MAPK kinase; MEF = murine embryonic fibroblast; MEK = mitogen-activated protein kinase, MEKK = MEK kinase; MMP = matrix metalloproteinase; NF = nuclear factor; P = phospho; sc, scrambled; siRNA, small interfering RNA; TAB = TAK1-binding protein, TAK = transforming growth factor-β activated kinase; TRAF6 = Tumor necrosis factor receptor-associated factor 6.

## Competing interests

The authors declare that they have no competing interests.

## Authors' contributions

DH designed and carried out experiments, DB made substantial contributions to the conception/design of the study and interpretation of data, TI helped design transfection experiments, GSF conceived of the study, participated in its design and coordination, and helped to draft the manuscript.
